# Multiple Origin but Single Domestication Led to *Oryza sativa*

**DOI:** 10.1534/g3.117.300334

**Published:** 2018-01-03

**Authors:** Jae Young Choi, Michael D. Purugganan

**Affiliations:** * Center for Genomics and Systems Biology, Department of Biology, New York University, New York 10003; † Center for Genomics and Systems Biology, New York University Abu Dhabi, Saadiyat Island, United Arab Emirates

**Keywords:** domestication, *Oryza sativa*, population genomics, admixture, phylogeny

## Abstract

The domestication scenario that led to Asian rice (*Oryza sativa*) is a contentious topic. Here, we have reanalyzed a previously published large-scale wild and domesticated rice data set, which was also analyzed by two studies but resulted in two contrasting domestication models. We suggest that the analysis of false-positive selective sweep regions and phylogenetic analysis of concatenated genomic regions may have been the sources that contributed to the different results. In the end, our result indicates that Asian rice originated from multiple wild progenitor subpopulations; however, *de novo* domestication appears to have occurred only once and the domestication alleles were transferred between rice subpopulations through introgression.

Asian rice (*Oryza sativa*) is a diverse crop comprising of five subpopulations (Garris *et al.* 2005). Archaeobotanical data for japonica and indica, the two major subpopulations of *O. sativa*, suggests that japonica was domesticated first, ∼7000 yr ago in the Yangtze Basin of China, while indica was domesticated later, ∼4000 yr ago in the Ganges plains of India (Fuller *et al.* 2010). Elucidating the origins of Asian rice and the history of its domestication has been a contentious field (Gross and Zhao 2014). With whole-genome data, it is becoming apparent that each Asian rice variety group/subspecies (aus, indica, and japonica) had distinct subpopulations of wild rice (*O. nivara* or *O. rufipogon*) as its progenitor (Huang *et al.* 2012). Specifically, wild rice could be divided into three major subpopulations and were designated as Or-I, Or-II, and Or-III by Huang *et al.* (2012). Phylogenetically, japonica was most closely related to Or-III, while aus and indica were most closely related to Or-I, but each was monophyletic with a different subset of Or-I samples (Huang *et al.* 2012). These results were consistent with a recent whole-genome study showing that distinct wild progenitors led to aus, indica, and japonica domestication (Choi *et al.* 2017).

Whether rice was domesticated once and subsequent varieties were formed by introgression with different wild progenitors, or whether each variety was domesticated independently in different parts of Asia, is debatable. The debate mainly arose from two studies analyzing the same data but surprisingly arriving at two different domestication scenarios: Huang *et al.* (2012) support the single domestication with introgression model, while Civáň *et al.* (2015) support the multiple domestication model. If the causal domestication mutation arose in a single genotype and was subsequently introgressed into the other two subpopulations (single domestication with introgression model), gene trees for the domestication region would differ from the genome-wide tree. Because the domestication is hypothesized to have occurred once in a single subpopulation and spread to the other subpopulations through hybridization, we define it as the single *de novo* domestication with introgression model. On the other hand, if the domestication mutation arose in each subpopulation on a different genetic background and was independently selected (multiple domestication model), then the gene trees for the domestication region would be concordant with the genome-wide tree. As domestication should leave evidence of recent selection, both studies used a reduction in polymorphism levels as a metric to detect local genomic regions associated with domestication. The evolutionary histories of those regions were then interpreted as the domestication history for Asian rice.

We argue that there may have been two issues that may have led to the conflicting results between Huang *et al.* (2012) and Civáň *et al.* (2015). First, regions that were detected by the two studies are candidate selective sweep regions that may or may not be related to domestication. Even population genetic model-based methods of detecting selective sweeps are prone to false positives, and with the right condition, any evolutionary scenario can be interpreted with a false-positive selective sweep region (Pavlidis *et al.* 2012). Because lineage sorting within a genomic region depends on the effective population size (N_e_) of that region (Pamilo and Nei 1988), evolutionary factors that decrease N_e_ (*e.g.*, population bottleneck, positive or negative selection, and low recombination rate) can accelerate the lineage sorting process (Hobolth *et al.* 2011; Prüfer *et al.* 2012; Scally *et al.* 2012; Pease and Hahn 2013). Given that each Asian rice had separate wild progenitor populations of origin, any false-positive selective sweep region will likely be concordant with the underlying species phylogeny and spuriously support the multiple domestication model. Hence, any candidate domestication-related selective sweep region would need additional evidence before being considered for downstream evolutionary analysis. Second, both studies used genotype calls made from low-coverage (1∼2×) resequencing data (Huang *et al.* 2012). Uncertainty associated with genotype calls made from low-coverage data (Nielsen *et al.* 2011) could be another source that led to the different results for the two studies.

Thus, we revisited the domestication scenarios proposed by the two studies and reanalyzed the Huang *et al.* (2012) data using a complete probabilistic framework that takes the uncertainty in SNP and genotype likelihoods into consideration (Fumagalli *et al.* 2014; Korneliussen *et al.* 2014). We then carefully compared our results against the two domestication models and contrasted it against the results from both the Huang *et al.* (2012) and Civáň *et al.* (2015) studies.

## Materials and Methods

Raw paired-end FASTQ data from the Huang *et al.* (2012) study was download from the National Center for Biotechnology Information website under bioproject ID numbers PRJEB2052, PRJEB2578, and PRJEB2829. We excluded the aromatic rice group from the analysis as the sample sizes were too small, and we excluded the few samples that had too high coverage. In the end, a total of 1477 samples were selected for analysis (Supplemental Material, Table S1).

Raw reads were then trimmed for adapter contamination and low-quality bases using trimmomatic ver. 0.36 (Bolger *et al.* 2014) with the command:

java –jar trimmomatic–0.36.jar PE \

$FASTQ1 $FASTQ2\

$FASTQ1_paired $FASTQ1_unpaired $FASTQ2_paired $FASTQ2_unpaired \

ILLUMINACLIP:TruSeq2–PE.fa:2:30:10:4 \

LEADING:3 TRAILING:3 SLIDINGWINDOW:4:15 MINLEN:30

Quality controlled FASTQ reads were then realigned to the reference japonica genome downloaded from EnsemblPlants release 30 (ftp://ftp.ensemblgenomes.org/pub/plants/). Reads were then mapped to the reference genome using the program BWA-MEM ver. 0.7.15 (Li 2013) with default parameters. Alignment files were then processed with PICARD ver. 2.9.0 (http://broadinstitute.github.io/picard/) and GATK ver. 3.7 (McKenna *et al.* 2010) toolkits to remove PCR duplicates and realign around INDEL regions (DePristo *et al.* 2011).

Using the processed alignment files, genotype probabilities were calculated with the program ANGSD ver. 0.913 (Korneliussen *et al.* 2014). The genotype probabilities were then used by the program ngsTools (Fumagalli *et al.* 2014) to conduct population genetic analysis. To estimate θ, ngsTools uses the site frequency spectrum as a prior to calculate allele frequency probabilities. Usually site frequency spectrum requires an appropriate outgroup sequence to infer the ancestral state of each site. However, for calculating Watterson and Tajima’s θ, it is not necessary to know whether each polymorphic site is a high- or low-frequency variant (Korneliussen *et al.* 2013). Hence, we used the same reference japonica genome as the outgroup, but strictly for purposes of calculating θ. Per site allele frequency likelihood was calculated using ANGSD with the commands:

angsd –b $BAMLIST –ref $REF –anc $REF –out $SFS –r $CHR \

–uniqueOnly 1 –remove_bads 1 –only_proper_pairs 1 –trim 0 \

–C 50 –baq 1 –minMapQ 20 –minQ 30 \

–minInd $minInd \

–setMinDepth $setMinDepth \

–setMaxDepth $setMaxDepth \

–doCounts 1 –GL 1 –doSaf 1

Per site allele frequency for each domesticated and wild subpopulation was calculated separately with different filtering parameters using the options –minInd, -setMinDepth, and -setMaxDepth; where parameter minInd represent the minimum number of individuals per site to be analyzed, setMinDepth represent minimum total sequencing depth per site to be analyzed, and setMaxDepth represent maximum total sequencing depth per site to be analyzed. Specifically, -minInd and –setMinDepth were set as one-third of the number individuals in the subpopulation, while –setMaxDepth was set as five times the number individuals in the subpopulation. Overall site frequency spectrum was then calculated with the realSFS program from the ANGSD package. Using each subpopulation’s site frequency spectrum as prior, we then calculated θ for each subpopulation using ANGSD with the command:

angsd –b $BAMLIST –ref $REF –anc $REF –out $THETA –r $CHR \

–uniqueOnly 1 –remove_bads 1 –only_proper_pairs 1 –trim 0 \

–C 50 –baq 1 –minMapQ 20 –minQ 30

–minInd $minInd \

–setMinDepth $setMinDepth \

–setMaxDepth $setMaxDepth

–doCounts 1 –GL 1 –doSaf 1 \

–doThetas 1 –pest $SFS

Using the output file from the previous command, for each subpopulation a sliding window analysis was then conducted with the thetaStat program from the ANGSD package using nonoverlapping window length and step sizes of 20, 100, 500, and 1000 kbp with the command:

thetaStat do_stat $ALLELEFREQ_POSTPROB_FILE \

–nChr $IND \

–win $Window –step $STEP

For each window, θ per site was estimated by dividing Tajima’s θ (θ_π_) against the total number of sites with data in the window. Windows with < 25% of sites with data were discarded from downstream analysis. This resulted in a minimum of 90% of the windows being analyzed (Table S2). Sweeps were identified using sliding windows that were estimating the ratio of wild to domesticate polymorphism (π_w_/π_d_). To calculate π_w_/π_d_ values, we chose the Or-II subpopulation to calculate π_w_, since the Or-II subpopulation was most distantly related to all three domesticated rice subpopulations (Figure S1). π_w_/π_d_ values were calculated separately for each domesticated rice subpopulation. Windows with large π_w_/π_d_ values were designated as candidate domestication selective sweep regions, and significance was determined using an empirical distribution of π_w_/π_d_ values described below.

Japonica has demographic history that is consistent with more intense domestication related bottlenecks then aus and indica (Xu *et al.* 2011). Thus, many π_w_/π_d_ values for japonica are expected to be similar between true domestication sweep and neutral regions, causing difficulties in identifying true-positive selective sweeps. Hence, we chose the approach of Civáň *et al.* (2015) by using a single threshold π_w_/π_d_ value to determine significance for all three subpopulations. In contrast to Civáň *et al.* (2015), we chose our threshold based on the empirical distribution of each subpopulation. The 97.5 percentile π_w_/π_d_ values were determined for each domesticated rice subpopulation, and the subpopulation with the lowest 97.5 percentile π_w_/π_d_ values was decided as the significance threshold. The threshold percentile that is represented by each subpopulation and window size is listed in Table S3. If the top 2.5% windows in the subpopulation with the lowest π_w_/π_d_ threshold are all due to true domestication sweeps, then in the other two subpopulations the threshold may be seen after a selective sweep or a population bottleneck. These colocated low-diversity genomic regions (CLDGRs) then represent candidate domestication-related selective sweep regions for all three subpopulations, and it is necessary for each CLDGR to have additional information to differentiate itself from the background domestication-related bottleneck scenarios. We assumed CLDGRs overlapping genes with functional genetic evidence related to domestication phenotypes (Meyer and Purugganan 2013) to be true candidate domestication genes. Custom R (R Core Team 2016) code for identifying the overlapping selective sweep windows can be found in the GitHub repository: https://github.com/cjy8709/Huangetal2012_reanalysis.

To account for the uncertainty in the underlying data, phylogenetic analyses were conducted by estimating pairwise genetic distances from genotype probabilities (Vieira *et al.* 2016). We ran the program ANGSD to calculate genotype probabilities for all 1477 domesticated and wild rice samples using the command:

angsd –b $BAMLIST –ref $REF –out $GENOPP –r $CHR \

–uniqueOnly 1 –remove_bads 1 –only_proper_pairs 1 –trim 0 \

–C 50 –baq 1 –minMapQ 20 –minQ 30 \

–minInd $minInd

–setMinDepth $setMinDepth

–setMaxDepth $setMaxDepth

–doCounts 1 –GL 1 –doMajorMinor 1 –doMaf 1 \

–skipTriallelic 1 –SNP_pval le–3 –doGeno 8 –doPost 1

Initially, the effects of different filtering parameters on the downstream phylogenetic analysis were examined by using three different parameter values for the options –minInd, -setMinDepth, and -setMaxDepth: (1) minInd = 492, setMinDepth = 492, setMaxDepth = 4920; (2) minInd = 738, setMinDepth = 738, setMaxDepth = 8862; and (3) minInd = 492, setMinDepth = 369, setMaxDepth = 8862. Afterward, all subsequent phylogenetic analyses were conducted with genotype posterior probabilities calculated using the minInd = 492, setMinDepth = 492, and setMaxDepth = 4920 parameter set. Genotype posterior probabilities were then used by the program ngsDist from the ngsTools package to estimate all pairwise genetic distances. Using the output file from the previous command, neighbor-joining trees were reconstructed with the genetic distances using the program FastME ver. 2.1.5 (Lefort *et al.* 2015) with the command:

fastme–2.1.5–linux64 –D 1 –i $DISTANCE_FILE –o $TREE

Phylogenetic trees were diagramed using the web interface iTOL ver. 3.4.3 (http://itol.embl.de/) (Letunic and Bork 2016).

### Data availability

The authors state that all data necessary for confirming the conclusions presented in the article are represented fully within the article. Custom R (R Core Team 2016) code for identifying the overlapping selective sweep windows can be found in the GitHub repository: https://github.com/cjy8709/Huangetal2012_reanalysis.

## Results and Discussion

**Figure 1:**
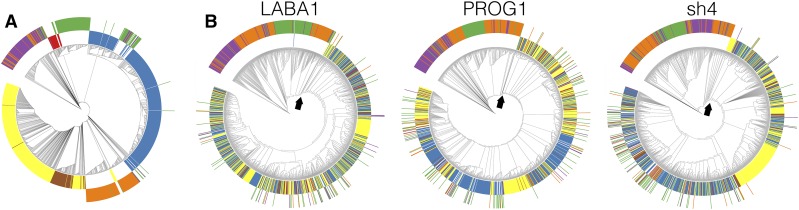
Neighbor-joining tree for (A) chromosome 1 and (B) 20 kbp upstream and downstream (40 kbp in total) of domestication genes *LABA1*, *PROG1*, and *sh4*. Inner circles of colors represent domesticated rice: red, aus; blue, indica; yellow, temperate japonica; and brown, tropical japonica. Outer circles of colors represent wild rice as designated by Huang *et al.* (2012): green, Or-I; purple, Or-II; and orange, Or-III. Arrows indicate the most ancestral internal node of the monophyletic domesticated rice clade.

In both of the studies by Huang *et al.* (2012) and Civáň *et al.* (2015), the phylogeny based on genome-wide data *vs.* putative domestication regions were compared to determine which domestication scenario was best supported by the data. We reconstructed the genome-wide phylogeny by estimating genetic distances between domesticated and wild rice using genotype probabilities (Vieira *et al.* 2016). To examine the effects that different parameters would have on downstream analysis, three different parameters were used to estimate the genotype probabilities. The probabilities were then subsequently used to estimate genetic distances and build neighbor-joining trees for each chromosome (Figure S1). Comparing trees built from the three different parameters, each chromosomal phylogeny was largely concordant with the others forming two major clades where the japonicas were grouping together, while indica and aus formed a monophyletic group (Figure 1A). Further, the trees corroborated the results of Huang *et al.* (2012), where the japonicas were most closely related to the Or-III wild rice subpopulation, while indica and aus were most closely related to the Or-I wild rice subpopulation.

We then scanned for local genomic regions associated with domestication-related selective sweeps to infer the domestication history of Asian rice. Compared to the π_w_/π_d_ method, there are population genetic model-based tests that are more powerful for detecting selective sweeps (Vitti *et al.* 2013). However, these methods use hard-called genotypes and do not take the uncertainty associated with low-coverage data into account. But more importantly, the two previous studies of Huang *et al.* (2012) and Civáň *et al.* (2015) used the π_w_/π_d_ method to detect domestication-related sweeps. For consistency we also implemented the π_w_/π_d_ method using genotype likelihoods to take the low coverage into consideration, and took a closer look at the genomic regions with significant evidence of a selective sweep.

To identify putative selective sweep regions, we chose the approach of Civáň *et al.* (2015) and identified sweep regions separately for each rice subpopulation. If rice had a single *de novo* domestication of origin in one subpopulation and the other two subpopulations were domesticated through introgression, then all three rice subpopulations would have identical sweep regions with shared haplotypes; otherwise, the single *de novo* domestication with introgression model cannot be supported. CLDGRs (Civáň *et al.* 2015) were identified using a window size that was different from both Huang *et al.* (2012) (100 kbp) and Civáň *et al.* (2015) (between 100 and 200 kbp), using a 20 kbp sliding window to narrow down on the candidate genes relating to domestication. To identify significant CLDGRs, we chose a stringent cutoff to conservatively identify candidate regions and identified a total of 39 CLDGRs (Table S4).

Neighbor-joining trees were then reconstructed for each of the 39 CLDGRs (Figure S2). The majority of CLDGRs showed monophyletic relationships among the domesticated rice subpopulation, where japonica, indica, and aus were clustering between and not within subpopulation types. Six windows (*e.g.*, chr2:11,660,000–11,680,000) showed phylogenetic relationships where each domesticated sample was clustering with the same domesticated subpopulation type. This initially suggested that the evolutionary history of CLDGRs was most consistent with the single *de novo* domestication model. We then examined larger window sizes of 100, 500, and 1000 kbp for candidate CLDGRs (Table S4) and reconstructed phylogenies for those regions (Figure S3, Figure S4, and Figure S5). Larger window sizes have fewer numbers of windows for analysis, hence leading to fewer numbers of CLDGRs being identified (Table S2). Nonetheless, with increasing window sizes, CLDGR phylogenies became more congruent with the genome-wide phylogenies, consistent with the multiple domestication model. However, CLDGRs are only candidate regions that may harbor domestication genes or may be false-positive selective sweep regions affected by domestication-related bottlenecks. As population bottlenecking can decrease effective population sizes, false-positive CLDGRs may represent regions of the genome with increased lineage sorting. These regions are then likely to have phylogenies that are more concordant with the underlying species phylogeny (Pamilo and Nei 1988). Hence, it is crucial that a CLDGR has additional evidence that can associate it with selection and differentiate its evolutionary history from the underlying species phylogeny. To do so, we searched CLDGRs that overlapped genes with functional genetic evidence related to domestication. We found three known domestication genes: long and barbed awn gene *LABA1* (chr4:25,959,399–25,963,504), the prostrate growth gene *PROG1* (chr7:2,839,194–2,840,089), and shattering locus *sh4* (chr4:34,231,186–34,233,221) (Li *et al.* 2006; Tan *et al.* 2008; Hua *et al.* 2015). Interestingly, the *sh4* gene was the only gene detected across multiple sliding window sizes, excluding the largest 1000 kbp window (Table S4).

Phylogenetic trees were then reconstructed for the three domestication loci that included 20 kbp upstream and downstream (40 kbp in total) of their coding sequence. We note for all three genes that the casual variants resulting in the domestication phenotype were located in the protein-coding sequences (Li *et al.* 2006; Jin *et al.* 2008; Hua *et al.* 2015). For all three genomic regions, the phylogenies were clustering different subpopulation types of domesticated rice together (Figure 1B), consistent with the single *de novo* domestication scenario.

**Figure 2:**
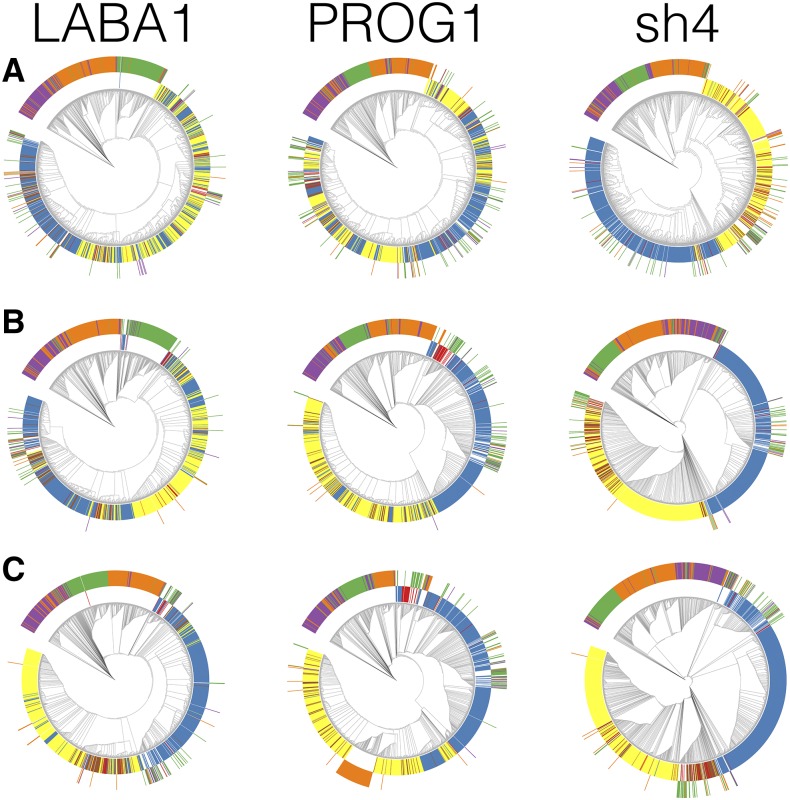
Neighbor-joining trees for three different window sizes flanking the domestication genes *LABA1*, *PROG1*, and *sh4*. (A) 50 kbp upstream and downstream of gene (100 kbp total); (B) 250 kbp upstream and downstream of gene (500 kbp total); and (C) 500 kbp upstream and downstream of gene (1000 kbp total).

Interestingly, *sh4* was identified as a candidate gene, with evidence of selective sweep in this study and both Huang *et al.* (2012) and Civáň *et al.* (2015). Only Civáň *et al.* (2015) did not find evidence of single origin in a phylogenetic tree reconstructed from a 240 kbp region surrounding *sh4*. A single-nucleotide mutation in the *sh4* gene causes a reduction in seed shattering (Li *et al.* 2006), which is an important domestication trait thought to minimize the labor during harvesting (Sang and Ge 2007). Sanger sequencing of the *sh4* region across various domesticated rice has indicated a near-identical haplotype at the *sh4* region, suggesting a single origin of nonshattering (Li *et al.* 2006; Zhang *et al.* 2009; Thurber *et al.* 2010). When we reconstructed phylogenies for 40 kbp windows surrounding the *sh4* region, the upstream region of the start codon had phylogenies where the domesticated rice were clustering with the same domesticated subpopulation types (Figure S6). We then reconstructed the phylogeny for large genetic regions surrounding each three domestication loci and discovered, with each increased window size, that the phylogeny of the region increasingly corroborated the genome-wide phylogeny by clustering with the same subpopulation type (Figure 2). This was not only true for *sh4*, but also *LABA1* and *PROG1* as well. Thus, the domestication-related evolutionary history for *sh4* is limited to the gene and regions that are downstream of the stop codon. Including large flanking regions or concatenating candidate domestication region without functional genetic evidence can lead to phylogenies that are concordant with the genome-wide species phylogeny, spuriously concluding it as evidence for the multiple domestication origin model. In fact, careful reexamination of the Huang *et al.* (2012) results indicates that the phylogeny for the concatenated 55 candidate domestication region actually shows a topology where japonica and indica were grouping with the same domesticated subpopulation type, while individual domestication regions with functional evidence showed a mix of japonica and indica clustering together, suggesting that concatenation had resulted in the neutral diversity of the nondomestication-related regions to overwhelm the phylogenetic signatures of the domestication regions.

In this study, we have used the same approach as Huang *et al.* (2012) and Civáň *et al.* (2015) to search for regions of domestication-related selective sweeps, and investigated those regions’ evolutionary history. With stringent thresholds and conservative assumptions to exclude false-positive CLDGRs, we were able to narrow down to three genes (*LABA1*, *PROG1*, and *sh4*), which were likely to be the key genes involved in the domestication of Asian rice (Meyer and Purugganan 2013). We note that our method of detecting selective sweeps may have missed other true domestication sweeps when other methods are applied (Liu *et al.* 2017), and the three genes may represent the minimum number of genes involved in the domestication of Asian rice. With higher coverage data becoming available (Li *et al.* 2014), powerful population genetic model-based selective sweep tests can be applied to detect more loci that were potentially involved in the domestication process. But even with these methods, one is still left with candidate regions and it is likely that different studies would detect different regions with significant evidence of undergoing a selective sweep, which could potentially result in contrasting domestication scenarios between studies. Hence, it is important that future domestication studies using advanced methods for detecting selection should consider whether the candidate region would also have functional genetic evidence as well.

Civáň *et al.* (2015) had criticized the role of *PROG1* and *sh4* in domestication due to several wild rice alleles clustering with the domesticated alleles (Figure 1B). However, evidence from dedomesticated weedy rice shows that feralized rice can carry the causative domestication allele but not retain any of the domestication phenotypes (Li *et al.* 2017), suggesting that some of the wild rice in the Huang *et al.* (2012) data set may actually represent different stages of feralized domesticated rice (Wang *et al.* 2017). Further, Civáň *et al.* (2015) claimed the clustering of wild and domesticated rice alleles as evidence of selection from standing variation (*i.e.*, soft sweep), which led to the observed phylogenies for *PROG1* and *sh4* (Civáň and Brown 2017). However, given the deep genome-wide divergence between the japonica and indica subpopulations (Choi *et al.* 2017; Wang *et al.* 2017), soft sweeps in this case are expected to produce distinct haplotypes and not homogenize the haplotypes between subpopulations (Messer and Petrov 2013). Thus, clustering of wild rice with domesticated rice in candidate domestication genes is more consistent with the frequent gene flow occurring between domesticated and wild rice (Wang *et al.* 2017). Subsequently, we caution the interpretation of phylogeographic analyses investigating the geographic localities of wild and domesticated CLDGRs, because phylogenetic clustering between wild and domesticated alleles cannot differentiate originating progenitor *vs.* recent hybridization between wild and domesticated rice.

Recently Wang *et al.* (2017) suggested that several wild rice samples in the Huang *et al.* (2012) study had evidence of gene flow from domesticated rice into the wild rice. Thus, the genetic affinity between the Or-I wild rice subpopulation and indica/aus, and the Or-III wild rice subpopulation and japonica, may represent recent gene flow, and it is unclear whether the Or-I and Or-III wild rice subpopulations represent the direct progenitor of domesticated rice. The deep genome-wide coalescence time between japonica and indica predating the archaeologically estimated domestication time (Choi *et al.* 2017) suggests that japonica and indica have independent wild rice of origin, but it is possible this progenitor was not sampled in the Huang *et al.* (2012) study. This is clearly seen across the genome-wide phylogeny of the domesticated rice as all samples cluster with the same domesticated subpopulation type. On the other hand, phylogenies from domestication loci were consistent with a single-origin model where all domesticated subpopulations were monophyletic with each other. Further, in all three regions, the most closely related wild rice corresponded to the Or-III subpopulation, supporting the hypothesis that the domestication alleles were introgressed from japonica into indica and aus (Huang *et al.* 2012; Choi *et al.* 2017). There is a possibility that the Or-III subpopulation, positioning as the sister group to all domesticated rice in domestication-associated genomic regions, may be an artifact from the gene flow between japonica and wild rice (Wang *et al.* 2017). However, we do not think this is the case for three reasons: (1) gene flow from domesticated to wild rice predominately originates from indica and aus subpopulations (Wang *et al.* 2017); (2) gene flow of domestication alleles into wild rice will cluster wild and domestication rice together, not position them as a separate sister group; and (3) even if it is a result of gene flow, the sister group position suggests that it was an old gene flow event ultimately involving the japonica subpopulation.

**Figure 3:**
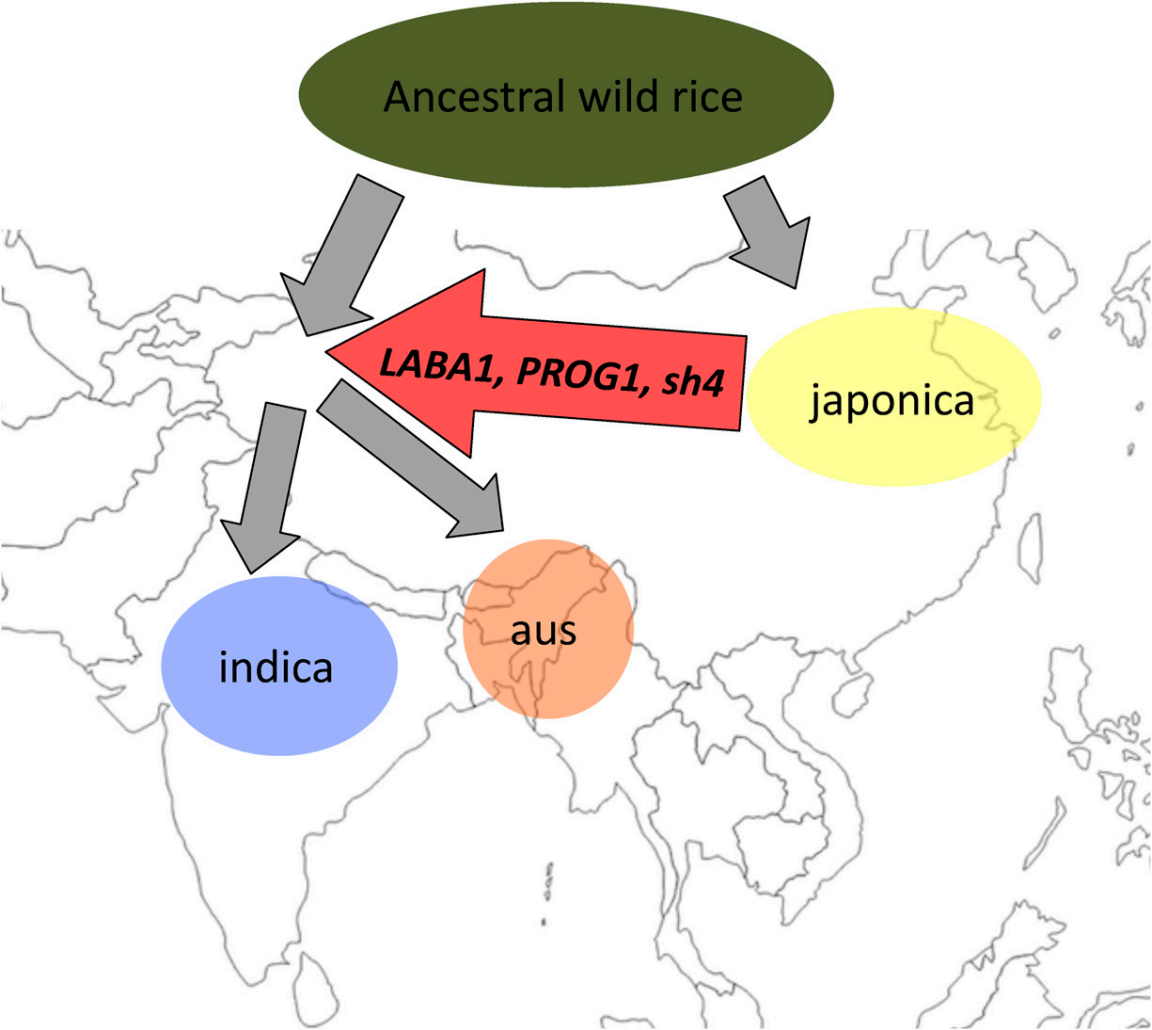
Domestication scenario that led to Asian rice. Each domesticated rice subpopulation had separate wild rice progenitor; however, because the geographic origin of the progenitor is heavily debated, its location is omitted from the map. Geographic position of the domesticated rice represents hypothesized domestication areas and was based on Fuller *et al.* (2010). Red arrow indicates a gene flow event that transferred the japonica originating domestication haplotypes from the genes *LABA1*, *PROG1*, and *sh4* into the progenitors of aus and indica, which led to their domestication.

In the end, our evolutionary analysis for the domestication loci *LABA1*, *PROG1*, and *sh4* are consistent with both Sanger and next-generation sequencing results (Li *et al.* 2006; Tan *et al.* 2008; Xu *et al.* 2011; Huang *et al.* 2012; Hua *et al.* 2015). Our results are also consistent with archaeological and genomic evidence (Fuller *et al.* 2010; Choi *et al.* 2017). Here then, we provide support for a model in which Asian rice has evolved from multiple origins but *de novo* domestication has only occurred once (Caicedo *et al.* 2007; Gao and Innan 2008; He *et al.* 2011; Huang *et al.* 2012; Castillo *et al.* 2016; Choi *et al.* 2017) (Figure 3). Specifically, this model hypothesizes that each domesticated rice subpopulation had a distinct wild rice subpopulation as its immediate progenitor, but the *de novo* domestication only occurred once in japonica, involving genes that include *LABA1*, *PROG1*, and *sh4*. The domestication alleles for these genes were then subsequently introgressed into the wild progenitors of aus and indica by gene flow, and ultimately led to their domestication. Indeed, crossing between wild and domesticated rice has been common practice in modern rice breeding for enhancing the limited domesticated rice genetic pool (Brar and Khush 1997), and may have been the ultimate source of diversifying the initial proto-domesticated rice into genetically differentiated domesticated rice subpopulations.

## Supplementary Material

Supplemental material is available online at www.g3journal.org/lookup/suppl/doi:10.1534/g3.117.300334/-/DC1.
