# Supplementary figures and images for: Multiple Origin but Single Domestication Led to Oryza sativa
Source: G3 (Bethesda). 2018 Jan 3;8(3):797–803. doi: 10.1534/g3.117.300334 (PMC5844301; doi:10.1534/g3.117.300334)

CHR1

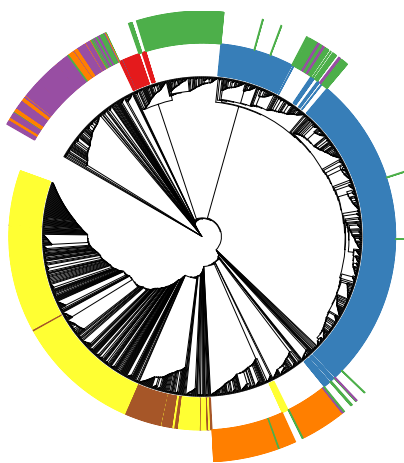

CHR2

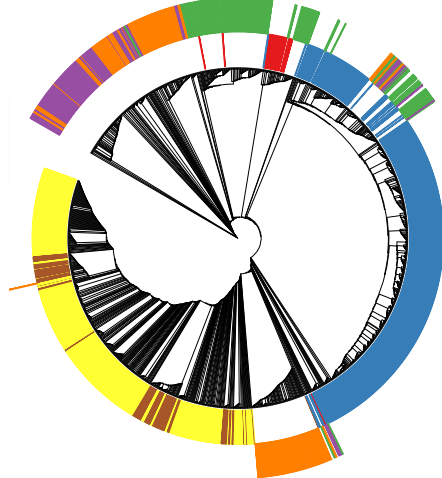

CHR3

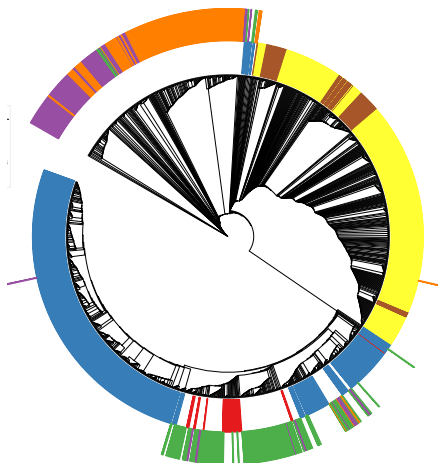

CHR4

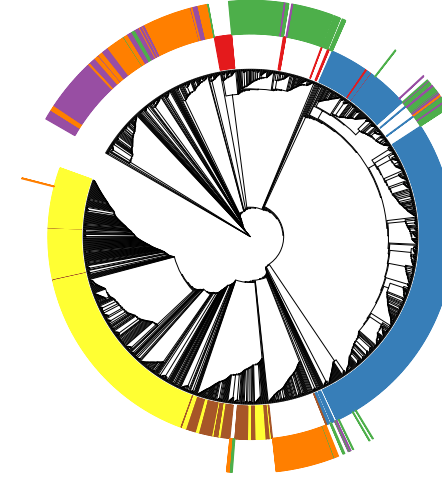

CHR5

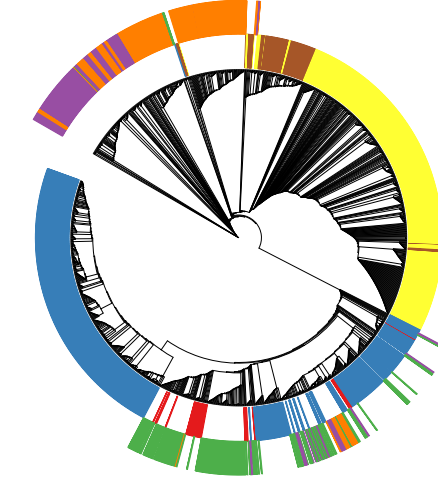

CHR6

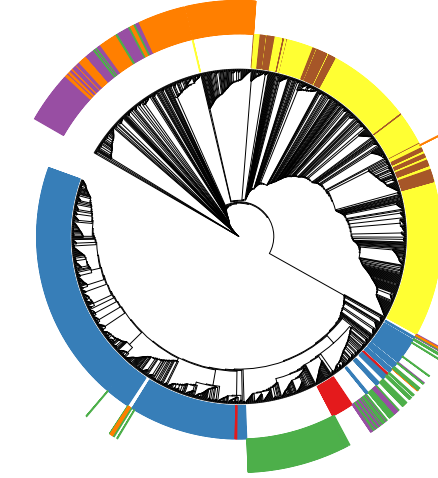

CHR7

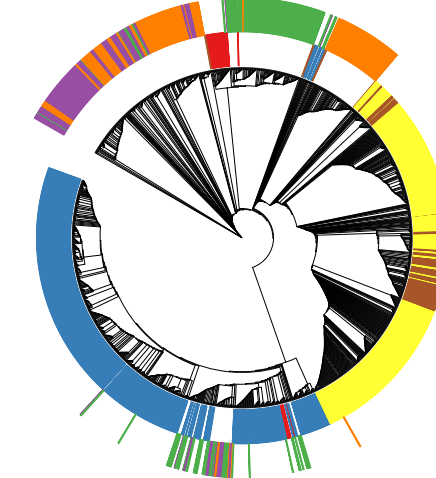

CHR8

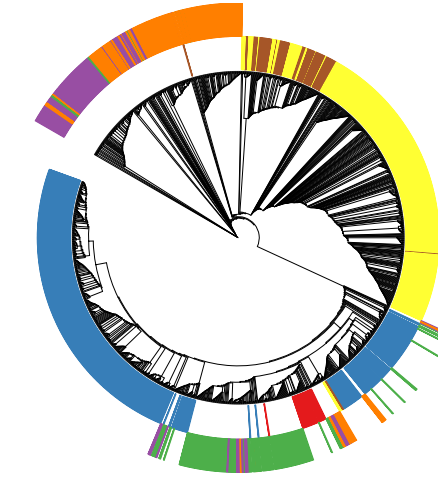

CHR9

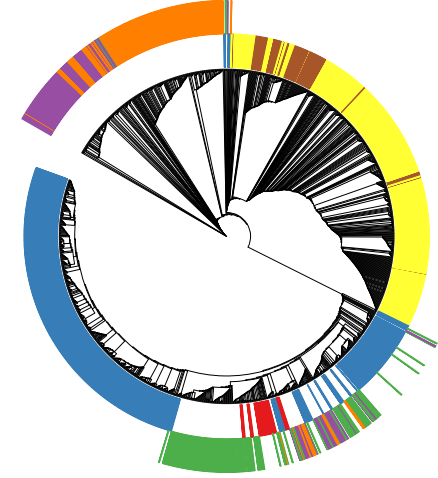

CHR10

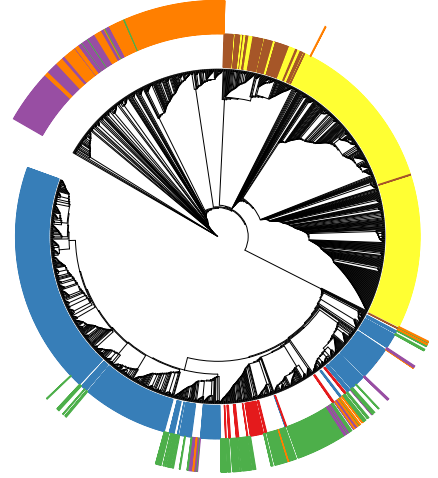

CHR11

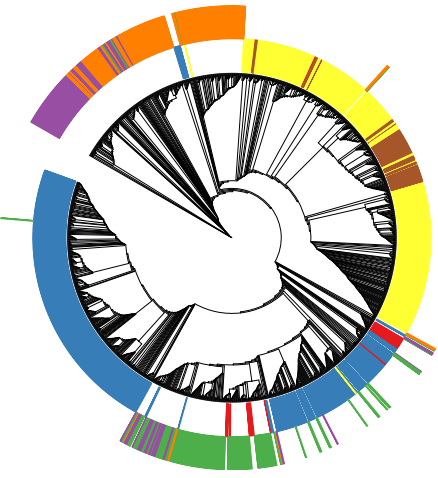

CHR12

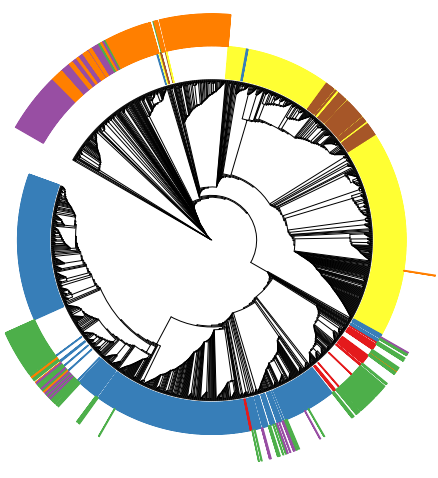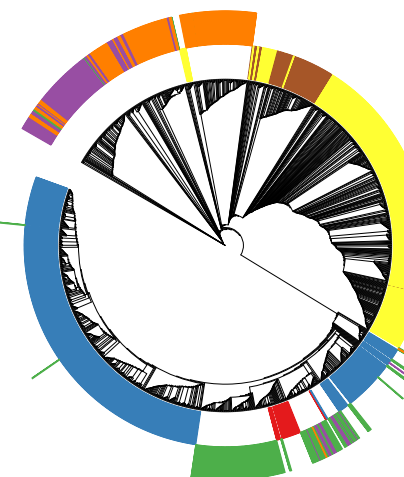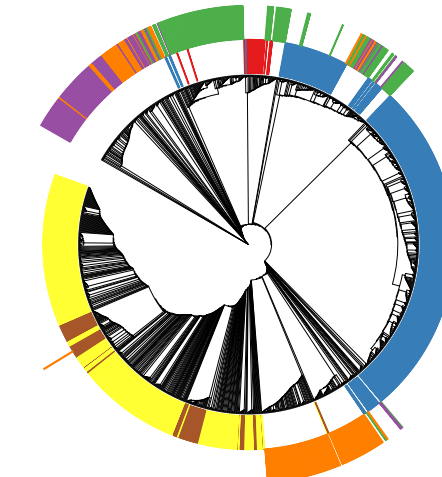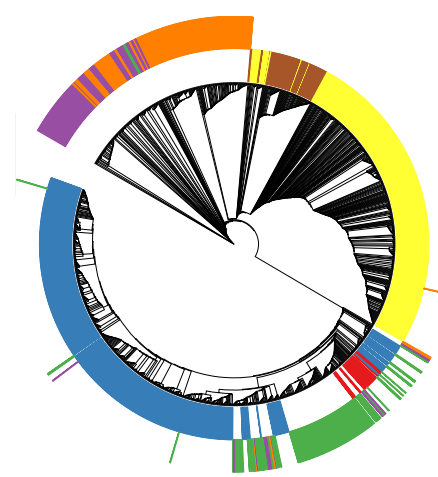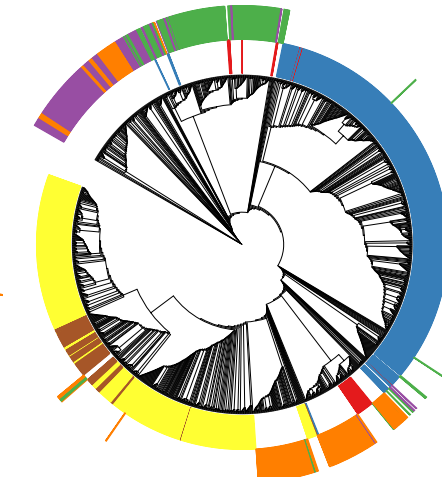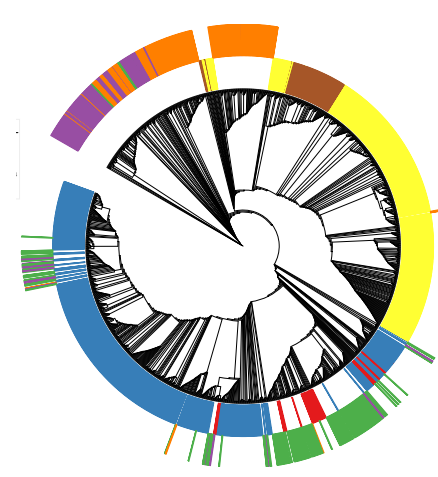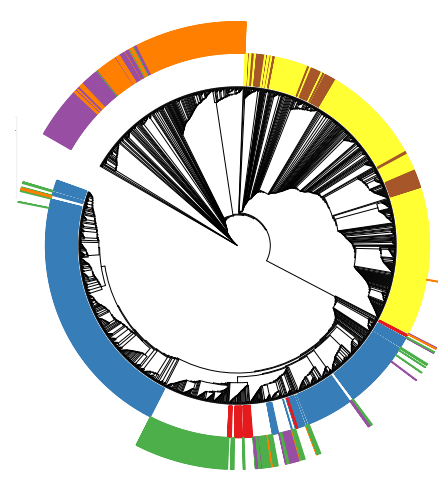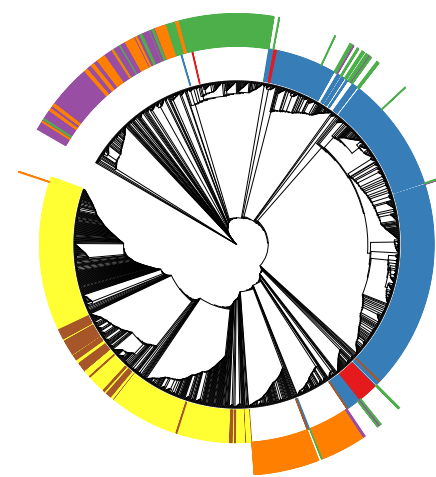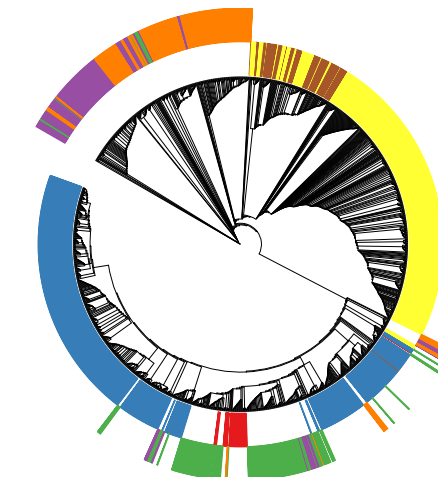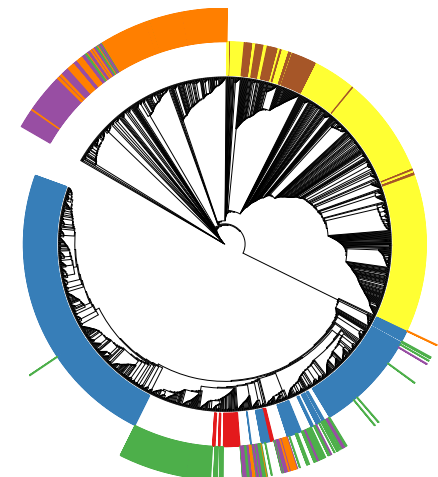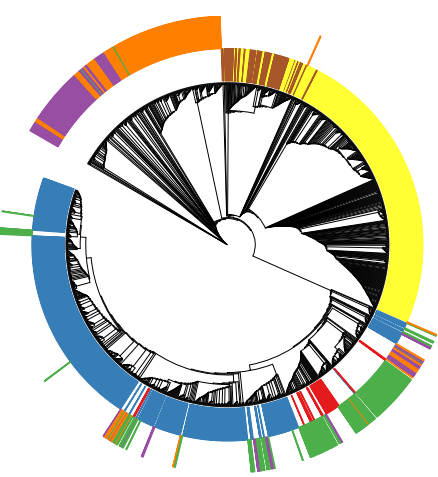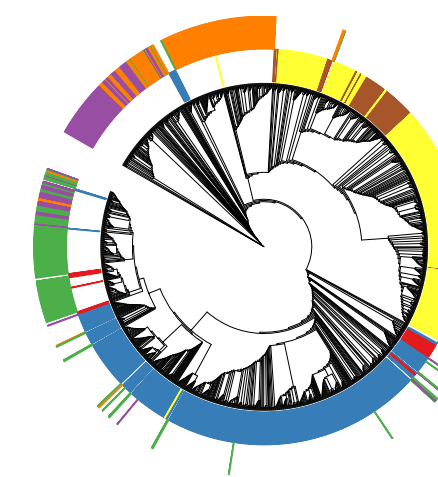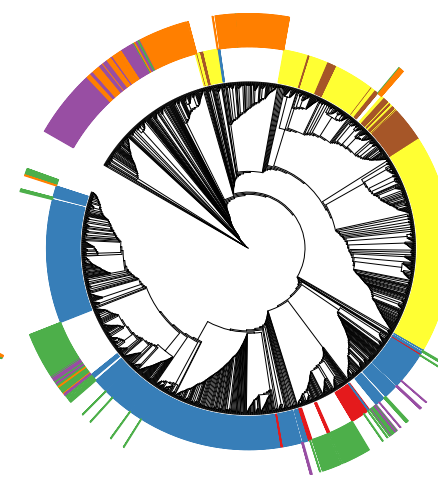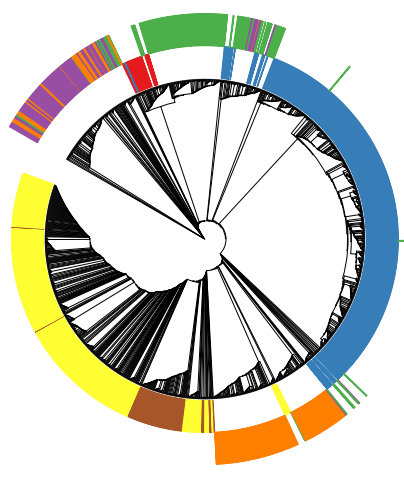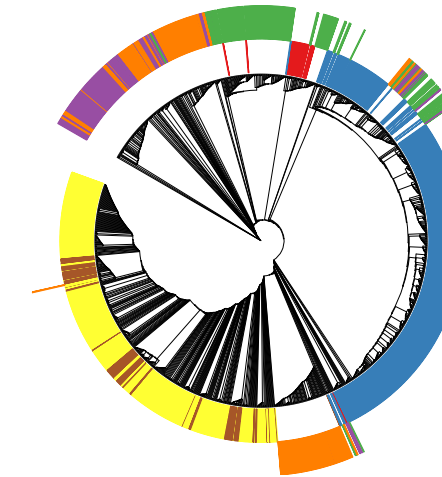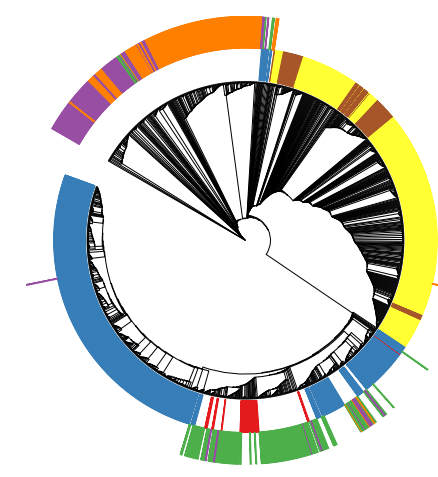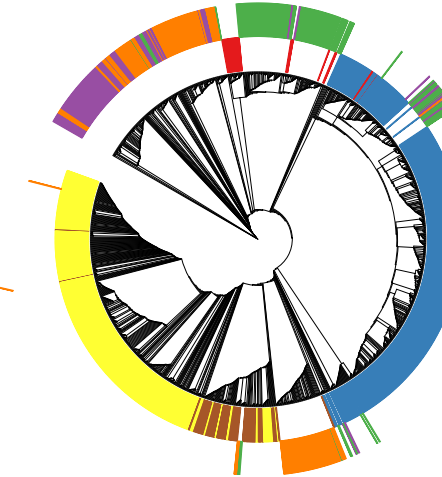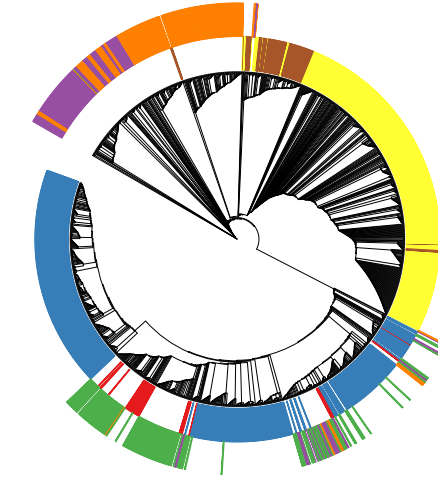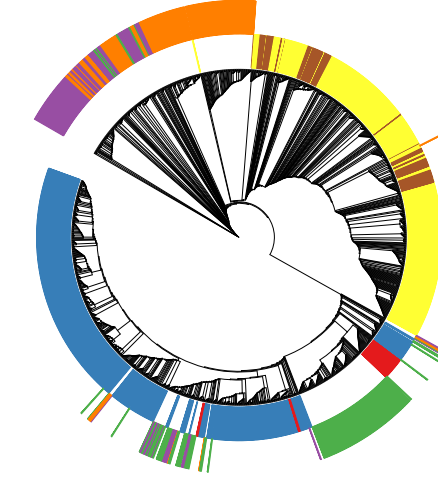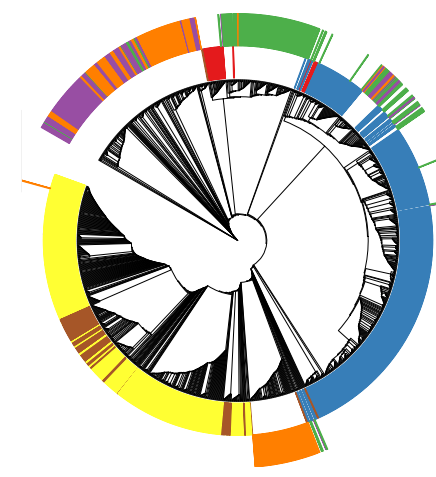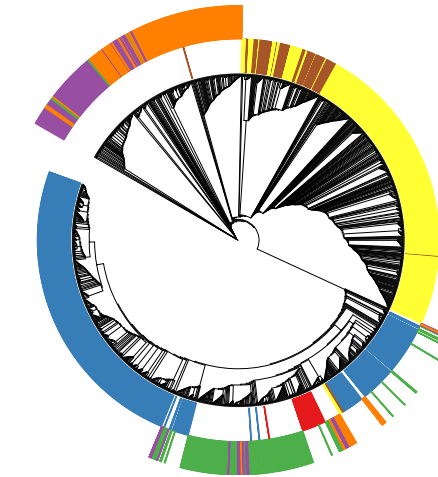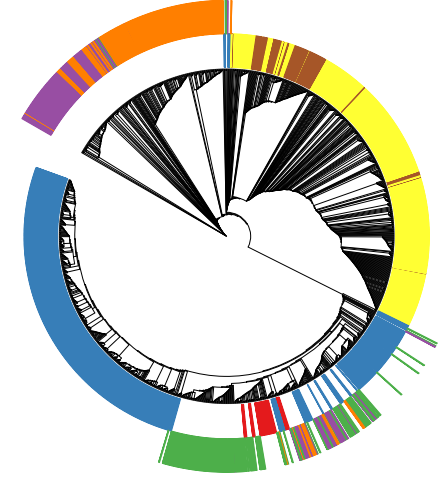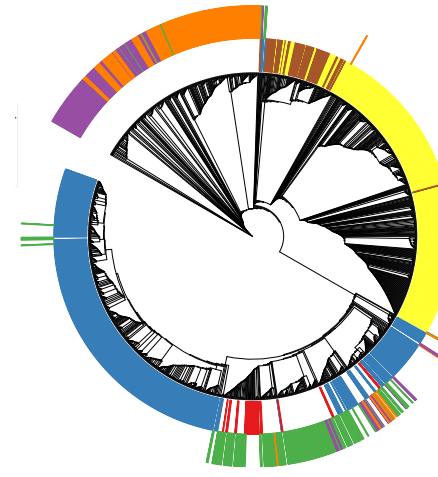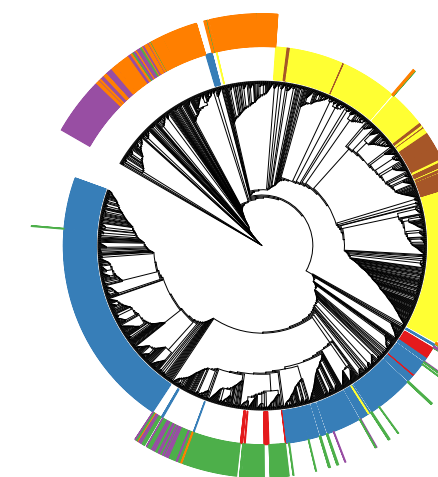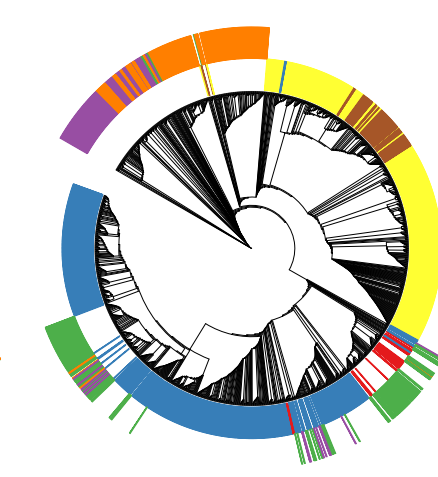

Supplement: Supplementary file 1 [file 797FigureS1.pdf]

2:1900000-1920000

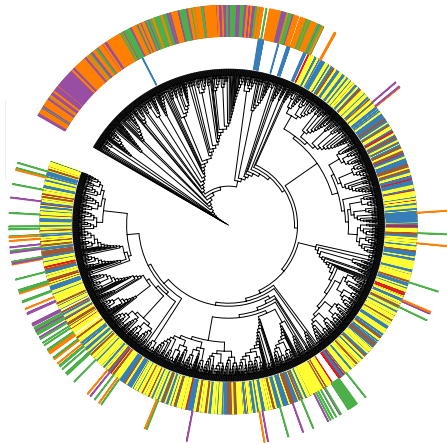

3:26180000-26200000

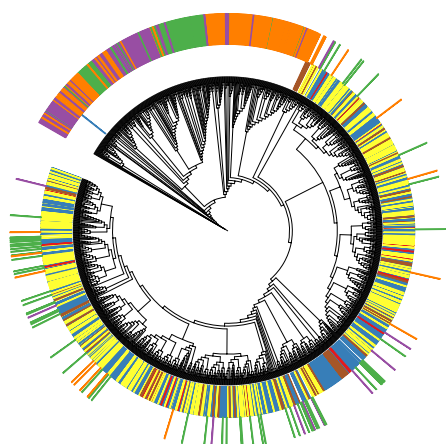

4:26080000-26100000

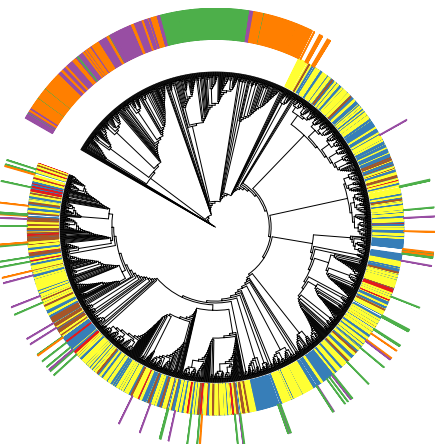

7:2740000-2760000

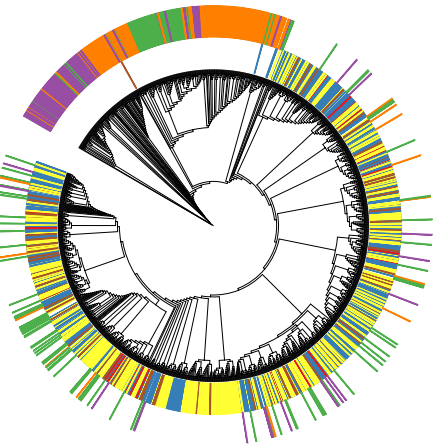

10:21340000-21360000

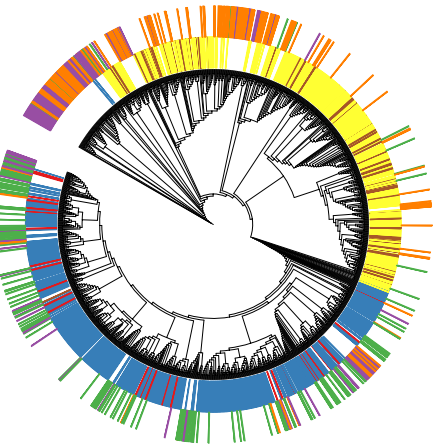

12:25380000-25400000

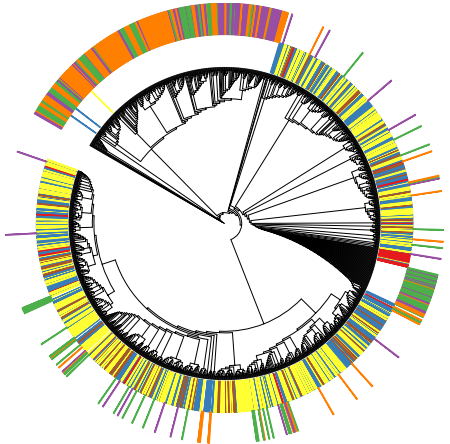

Supplement: Supplementary file 2 [file 797FigureS2.pdf]

1:8300000-8400000

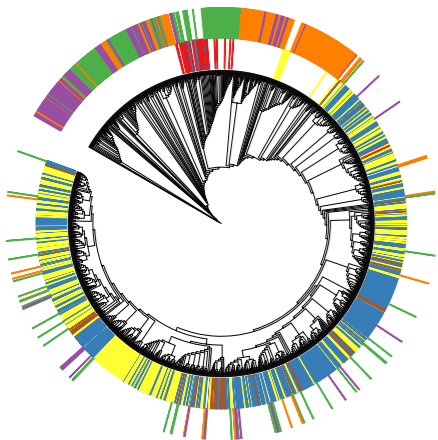

3:26100000-26200000

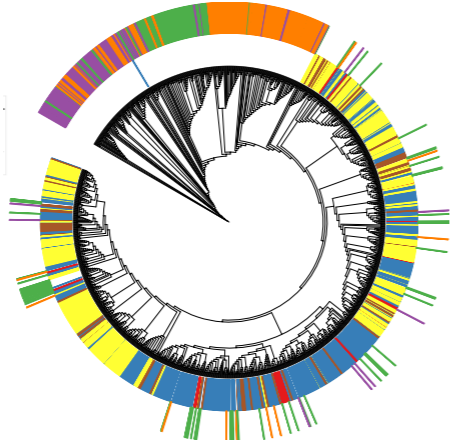

4:25800000-25900000

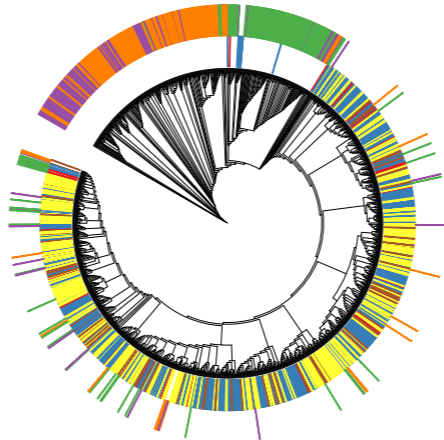

4:34100000-34200000

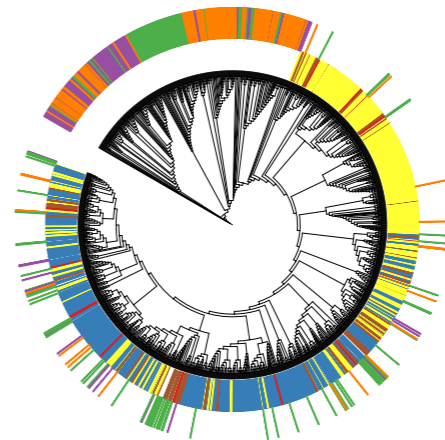

4:34200000-34300000

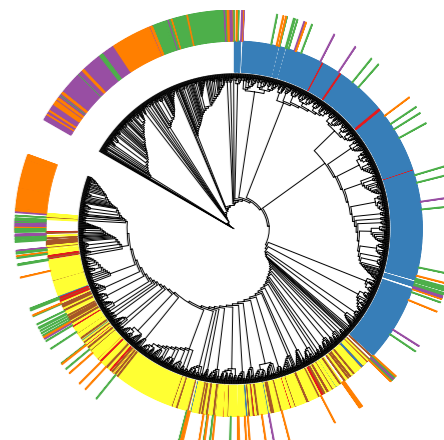

7:4000000-4100000

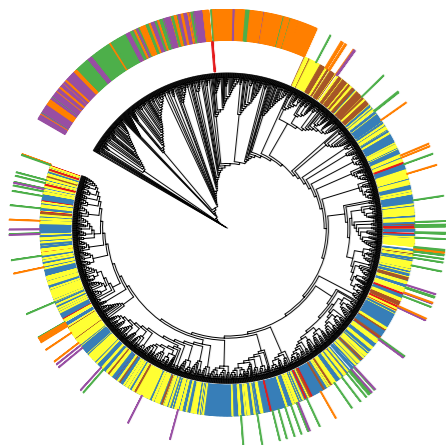

7:28300000-28400000

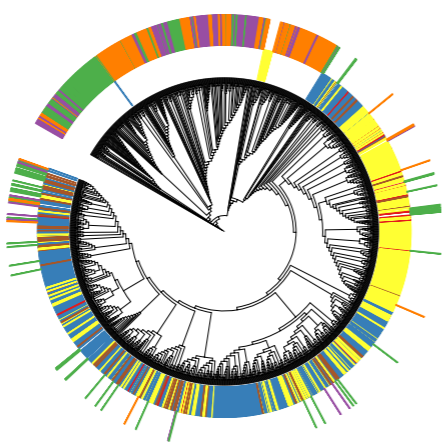

8:23800000-23900000

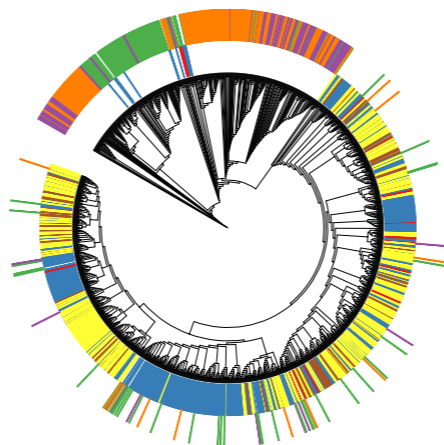

11:1400000-1500000

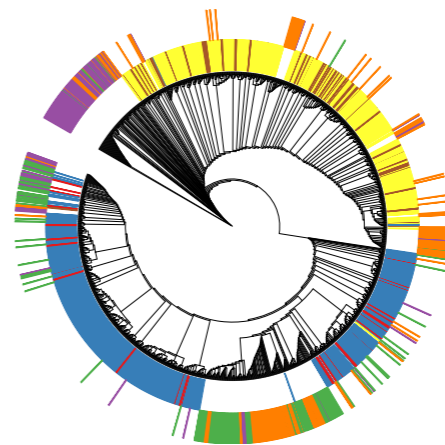

12:24900000-25000000

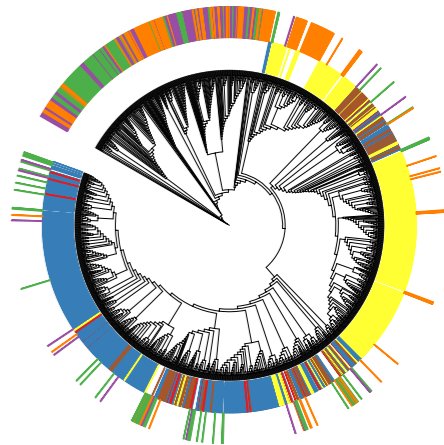

Supplement: Supplementary file 3 [file 797FigureS3.pdf]

4:34000000-34500000

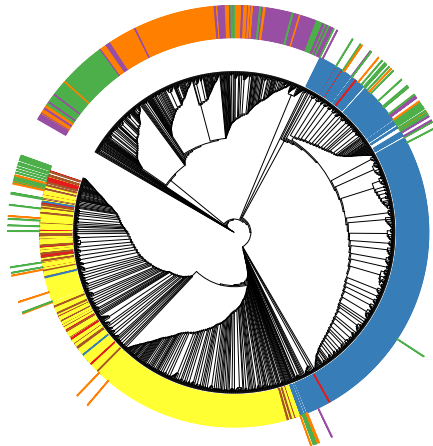

7:40000000-45000000

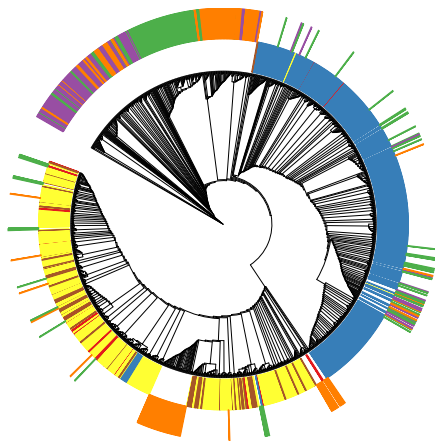

8:235000000-240000000

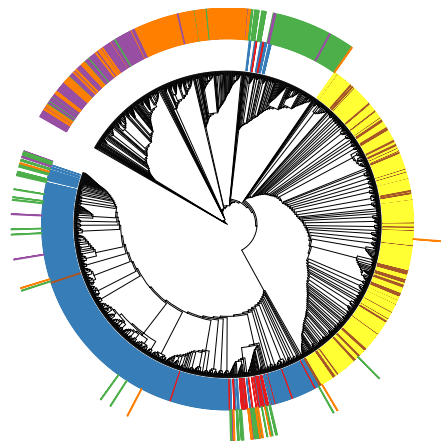

10:175000000-180000000

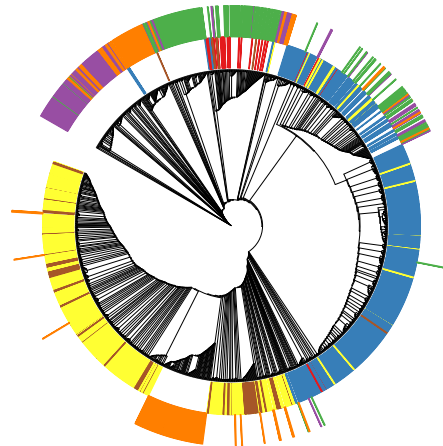

Supplement: Supplementary file 4 [file 797FigureS4.pdf]

2:120000000-130000000

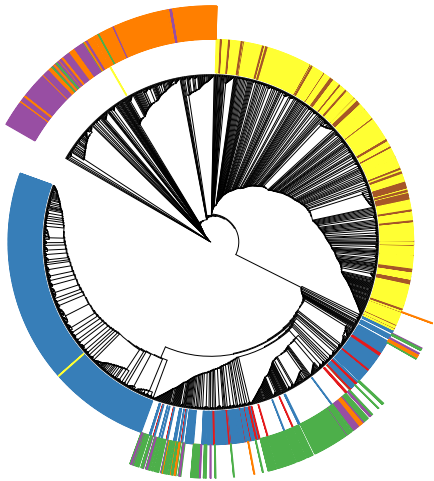

8:230000000-240000000

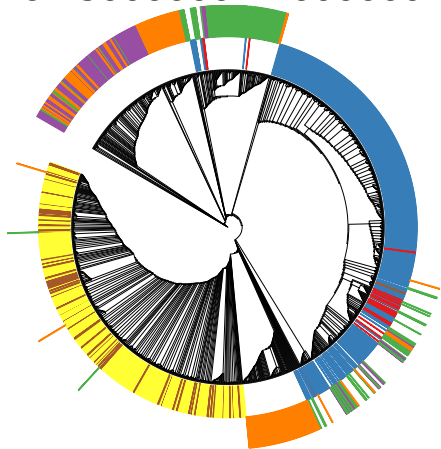

Supplement: Supplementary file 5 [file 797FigureS5.pdf]

4:34130000-34170000

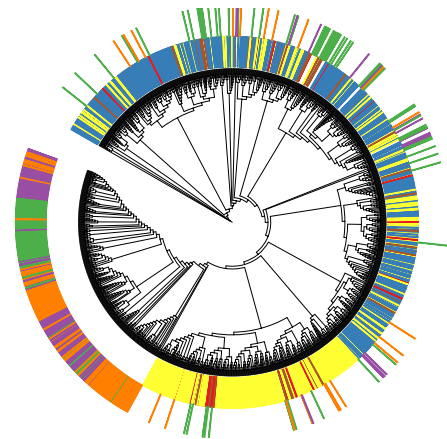

4:34170000-34210000

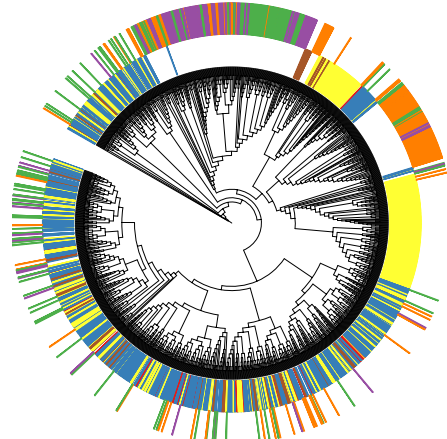

4:34210000-34250000

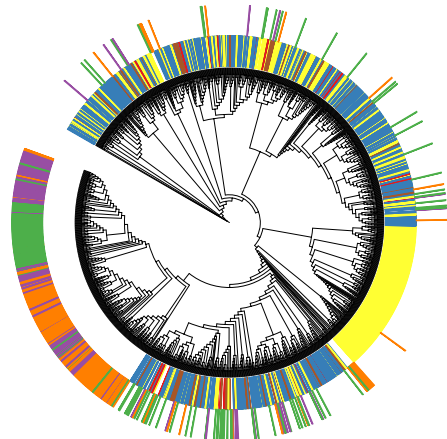

4:34250000-34290000

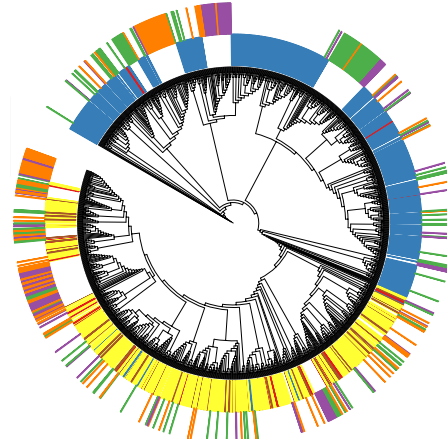

4:34290000-34330000

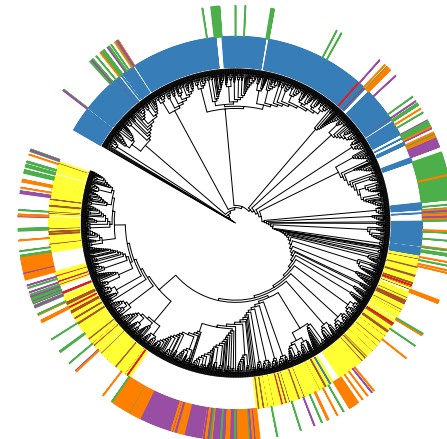

Supplement: Supplementary file 6 [file 797FigureS6.pdf]
